# The Heterogeneity of Longitudinal Cognitive Decline in Euthymic Bipolar I Disorder With Clinical Characteristics and Functional Outcomes

**DOI:** 10.3389/fpsyt.2021.684813

**Published:** 2021-07-21

**Authors:** Wen-Yin Chen, Ming-Chyi Huang, Ya-Chin Lee, Chiao-Erh Chang, Shih-Ku Lin, Chih Chiang Chiu, Hsing-Cheng Liu, Chian-Jue Kuo, Shih-Han Weng, Po-Yu Chen, Po-Hsiu Kuo

**Affiliations:** ^1^Department of Psychiatry, Taipei City Psychiatric Center, Taipei City Hospital, Taipei, Taiwan; ^2^Institute of Epidemiology and Preventive Medicine, College of Public Health, National Taiwan University, Taipei, Taiwan; ^3^Department of Psychiatry, School of Medicine, College of Medicine, Taipei Medical University, Taipei, Taiwan; ^4^Department of Education and Research, Taipei City Hospital, Taipei, Taiwan; ^5^Department of Public Health, College of Public Health, National Taiwan University, Taipei, Taiwan; ^6^Department of Psychiatry, National Taiwan University Hospital, Taipei, Taiwan

**Keywords:** BAC-A, bipolar disorder, function assessment, heterogeneity, longitudinal

## Abstract

We characterized the heterogeneity and risk factors of cognitive decline in euthymic bipolar disorder (BD), and their magnitude of associations with subjective daily functions. In this retrospective cohort, BD type I patients (*N* = 128) were followed for an average of 6.5 years. Intelligence quotient (IQ) at index date was recorded, and premorbid IQ was estimated. We used Brief Assessment of Cognition in Affective Disorders (BAC-A) to assess cognition at follow-up. We evaluated current functions with World Health Organization Disability Assessment Schedule 2.0. Clinical and sociodemographic factors were examined for their independent effects on longitudinal cognitive decline. In addition, we employed multivariate adaptive regression spline to detect inflection points for the nature of slope changes in cognitive decline among BD patients. During follow-up years, 21 BD patients (16.4%) showed longitudinal cognitive decline. In cognitive decline group, all cognitive domains of BAC-A were significantly worsened. We found that density of episodes with psychotic features was an independent risk factor for cognitive decline after adjusted for age, gender and dose of mood stabilizer. After the age of 42 years, a steeper cognitive change was observed in the cognitive decline group. The correlation pattern between cognitive domains and functional outcomes differed between patients with and without cognitive decline. The present study characterized cognitive heterogeneity longitudinally in BD patients. As density of episodes play roles for cognitive decline, our results emphasize the importance of relapse prevention. Our findings provide hints for future personalized interventions and facilitating genetic and biological studies for dissecting the heterogeneity of bipolar illness.

## Introduction

Bipolar disorder (BD), a recurrent chronic disorder, is characterized by episodes of mania and depression interspaced by euthymia, and it affects different aspects of daily living (1). BD is also associated with cognitive deficits in a number of domains, which may persist in patients in remission (2). For example, Robinson and Ferrier reported that euthymic patients with BD showed cognitive impairments compared with healthy controls, particularly in executive function and verbal learning (3). A meta-analysis using individual patient data further confirmed significant impairments in a wide-range of cognitive domains in BD. In particular, verbal learning test, executive function and processing speed showed robust impairments even controlling for age, educational years, gender, residual mood symptoms, and medications (4). Cognitive deficits have substantial negative impacts on social functioning and are responsible for poor inter-episode recovery or poor quality of life in a high proportion of patients (5–7), suggesting the importance of studying cognitive functions in BD to enhance future clinical care and prognosis outcomes.

The presence of cognitive impairment can regard as a marker of neuroprogression in BD patients (8). The underlying neurobiological mechanisms may include high pro-inflammatory activity, reduced neurotrophic support, and high oxidative stress burden in BD (9). Other factors such as higher rate of medical comorbidity, unhealthy related behaviors, and substance abuse (10, 11) also contribute to lower brain and cognitive reserve, thereby increasing risk of cognitive declines. In addition, recent studies reported substantial genetic overlap between cognitive function and BD risk (12).

On the other hand, previous cross-sectional studies have examined clinical factors with cognitive deficits in BD. Although findings were inconsistent, some studies have reported the correlations of cognitive impairments with illness severity (4). However, it is difficult to infer the causal link between cognitive deficits and clinical features due to the cross-sectional nature of these studies. One early study adopted a first-episode design to compare first-episode BD, schizophrenia, and healthy controls, and found that cognitive deficits are evident right from the first mood episode (13). During the disease course, cognitive impairment in BD patients then varies, with some studies suggesting that cognitive deficits stabilize over time (14–16), whereas others have shown a pattern of progressive deterioration (17, 18) and even increased risk of dementia in later life (19). It is believed that heterogeneity is widely observed among BD patients in this regard, in terms of the impaired cognitive domains, longitudinal cognition stability, and the speed of deteriorative progression. Longitudinal study design is preferable to explore cognitive declines over the course of bipolar illness, though it was relatively scarce in the literature.

So far, the heterogeneity of longitudinal cognitive decline in BD patients is not well-understood. First, insufficient data were available to investigate all cognitive domains in few longitudinal studies, and findings on deficits among different cognitive domains were inconsistent. Whether these are discrete areas of impairment or reflect an underlying single, more basic cognitive abnormality is as yet unclear. Second, previous longitudinal studies often consisted of small sample sizes and different follow-up periods, resulted in various findings. For example, the synthesis of longitudinal evidence suggests that the cognitive path of individuals with BD may be set early on and may not deteriorate over time (20, 21). However, another study with a much longer follow-up period showed significant test–retest differences in executive measures revealing decline (22), which implied the existence of cognitive instability over longer disease course. Third, the differences in medication variables or mood scales between assessment times could not be well-controlled in every study and may have influenced the results. In addition, we should consider the effect of repeated testing, wherein the true decline of cognitive functioning would be partially masked by learning effects during short period of follow-up.

We hypothesized that both the clinical course and cognitive decline are heterogeneous among BD patients, and thus may partially account for the inconsistency of findings in previous reviews. The majority of cross-sectional studies have suggested that 38–40% of patients with BD have no neurocognitive deficits, and 20–30% had obvious cognitive deficits (23–25), while less data on longitudinal cognitive decline. The complexity of cognitive impairment in BD may include neurodevelopmental, neuroprogressive, or combination of the two. The lack of longitudinal assessment of cognitive performance in BD hinders to explore the heterogeneity in this topic.

We aim to establish a retrospective cohort of bipolar disorder patients and to evaluate their cognitive changes over time by looking at age hinge points of cognitive decline, to characterize heterogeneous cognitive profiles, and to examine the risk factors for longitudinal cognitive decline. Lastly, we further evaluate whether different cognitive changes impact daily functions in patients with and without cognitive decline.

## Materials and Methods

### Participants

In this retrospective cohort, individuals who were diagnosed with BD subtype I according to Diagnostic and Statistical Manual of Mental Disorders, fourth Edition, Text Revision (DSM-IV-TR) by board-certified psychiatrists from outpatient clinics were invited to join this study between July 1, 2018 to the end of 2019 (study entry). They were more than 20 years of age, and we excluded (a) known substance use disorder (except nicotine use disorder); (b) any disorder with known neurological symptoms or complications such as brain injury or stroke; (c) a previous diagnosis of intellectual disabilities, schizophrenia or schizoaffective disorders; or (d) inability to complete the standard clinical assessment or providing informed consent. The information for psychiatry comorbidities and exclusion criteria is obtained through medical diagnosis record and by the Chinese version of the modified Schedule of Affective Disorder and Schizophrenia-Lifetime (SADS-L), which has been report to have high interrater reliability values of mood disorders (major depression: 0.79; bipolar disorder: 0.71) (26). Individuals were also required to be euthymic at the time of study entry and under stable medications (no change of medications in previous 1 month) for current cognitive assessment. To evaluate cognitive deficits, eligible participants who had a prior index intelligence quotient (IQ) testing during euthymic state in medical record were retained in the following analysis. Mood symptoms were obtained through clinician-administered measures of the 17-item Hamilton Depression Rating Scale (HDS) and Young Mania Rating Scale (YMRS). For euthymic state, we defined HDS and YMRS scores ≤ 8 within 7 days before assessment (27, 28).

### Measurements

#### Demographic Data, Clinical Course, and Index IQ

Patients' demographic and clinical course data were collected when study entry, from medical records and interviews by psychiatrists, if required. The clinical characteristics of participants included the number of affective episodes (total, manic, and major depressive), number of episodes with psychotic features, number of hospitalizations, age at illness onset, maximum length of free intervals, and number of suicide attempts. Adverse childhood events were assessed using Chinese version of Childhood Trauma Questionnaire-Short Form (CTQ-SF) (29). Considering the duration of illness varied across individuals, we also calculated episode density by dividing number of episodes by duration of illness as the total episode density, manic episode density, major depressive episode density and episode with psychotic features density separately. Psychopharmacological treatments used at the time of study entry were recorded and then transformed to defined daily dose (DDD). DDD is a unit of measurement assumed average maintenance dose per day for a drug used for its main indication in adults, which can be derived for comparisons of drug consumption (30, 31). Through reviewing medical records, we retrospectively recorded physical comorbidities, anthropometry, and index IQ at the euthymic state from the first clinical visit or admission of BD diagnosis. The index IQ was measured by a licensed psychologist using the Wechsler Adult Intelligence Scale (WAIS-III or WAIS-IV) in BD euthymic state and was regarded as a stable trait in adults. It was routinely assessed for the clinical practice in the recruitment hospital for assisting attending psychiatrists to establish proper rehabilitation plans for patients. In addition, we collected patients' premorbid educational years and occupational status with age and gender to estimate premorbid IQ (32, 33). Therefore, the cognitive information using IQ from medical records was defined at two time-points: T1, the premorbid estimated IQ; T2, the index IQ after BD onset (Details were illustrated in Supplementary Figure 1).

#### Current Cognitive Measurements

Enrolled patients were assessed using the Brief Assessment of Cognition in Affective Disorders (BAC-A) during their euthymic state for at least 1-year interval apart from the date of index IQ measure to ensure evaluating longitudinal change and minimize possible learning effects. The time point of BAC-A assessment was defined as T3 as study entry (Supplementary Figure 1). BAC-A has extensively been used as a rapid and reliable measure of cognitive assessment in a range of clinically affective patients (34). It takes ~35 min to administer. It provides measure of affective memory and emotional inhibition (Affective Processing Tests [APTs]) from Affective Auditory Verbal Learning Test and Emotional Stroop Task, and also measure for six traditional neurocognitive domains, namely, working memory (Digit Sequencing Task), motor speed (Token Motor Task), verbal fluency (Category Instances and Controlled Oral Word Association Test), attention and processing speed (Symbol Coding), verbal memory (List Learning), and executive function (Tower of London) with comparable norm references (35, 36). APTs were further applied with the indexes of Affective Interference Test (AIT), Emotion Inhibition Test (EIT), Delayed Recognition (DR), and Emotion Inhibition Index (EII) (37). The criterion and construct validity of each test for cognitive impairment as well as the sensitivity of these tests to changes in cognition have been demonstrated in the scientific literature, and each test has also been shown to be valid for use in different cultures and language groups (38).

#### Functional Assessment

Trained interviewer administered the World Health Organization Disability Assessment Schedule 2.0 (WHODAS 2.0) for multidimensional assessment of subjective function in the participants also in the time of study entry (T3). WHODAS 2.0 is a practical, generic assessment instrument that can measure health and disability at a population level or in clinical practice. It evaluates subjective function in six domains: Domain 1: cognition– understanding and communicating; Domain 2: Mobility– moving and getting around; Domain 3: Self-care– attending to one's hygiene, dressing, eating, and staying alone; Domain 4: Getting along– interacting with other people; Domain 5: Life activities– domestic responsibilities, leisure, work, and school; and Domain 6: Participation– joining in community activities and participating in society (39). The raw score is transformed into item response theory (IRT) scoring (0–100), with higher scores indicating increasing severity of subjective function impairment (39).

### Statistical Analysis

We explored cognitive heterogeneity in BD longitudinally and defined subjects dichotomously into groups with or without cognitive decline. We transformed cognitive performance into Z1, Z2b and Z3 scores for the estimated premorbid IQ at T1, index IQ at T2 and BAC-A composite score at T3, respectively. This transformation was done based on the same outside reference from standardization samples in Taiwan (33, 36, 40), to make different assessments comparable at each time point. First, we compared the cognitive performance between T1 and T2. Among 128 subjects, there were 23 who showed cognitive deficits comparing premorbid Z1 and Z2 at disease index date, while 105 retained their cognitive function at disease index date (having <2 standard deviations [SD] of cognitive changes from T1 to T2). Second, there were 21 revealed longitudinally cognitive decline (16 from 105 and 5 from 23, having > 2 SD cognition downward changes from T2 to T3), and 89 were classified into the cognitive non-declined group who had <2 SD cognition changes from T2 to T3. Finally, there remained 18 patients (stable cognitive deficits) who showed cognitive deficits at T2 with cognitive functions stable over the follow-up period at T3. In the following analyses, we compared BD groups with (*N* = 21) and without longitudinal cognitive decline (*N* = 89) only.

We compared demographic and clinical characteristics between BD patients with and without cognitive decline using the chi-square and Student's *t-*test for categorical and continuous variables, respectively. The differences in BAC-A and functional outcomes were also examined. Normality of data was assessed using the Kolmogorov-Smirnov test. For non-normally distributed data, we used non-parametric Wilcoxon rank sum test for analysis. The variables that showed significant differences between the two groups were further assessed by univariate logistic regression to reveal their influences on cognitive outcome. The significant factors from univariate analysis were then included in multivariable logistic regression with stepwise selection to evaluate their independent effects, while adjusted for age and gender. In the multivariable regression analyses, we excluded variables with high correlation with others to avoid multicollinearity in the model (e.g., correlation coefficient between manic episode density and density of episodes with psychotic features was high, equals to 0.59). Pearson correlations between specific cognitive and functional domains were demonstrated in each group. Moreover, multivariate adaptive regression spline (MARS) (41) and scatterplot with Loess curve were used to detect hinges or inflection points to characterize the timing and nature of slope changes in the different cognitive groups. MARS is a non-parametric regression technique that can model non-linearities, which is much suitable for detecting the hinge points (42). MARS analysis was conducted using the earth package in R (43); all other analyses were conducted using SAS (version 9.4; SAS Institute Inc.; Cary, NC, USA). There were very few missing numbers in each different variable, and were treated as missing data in all analysis. Significance level was set at *p* < 0.05.

## Results

### Patient Characteristics

In our retrospective cohort study, BD-I patients demonstrated longitudinal heterogeneous course in terms of cognitive functions, who presented with stable cognitive deficits (14.1%), with longitudinal cognitive decline (16.4%) and without cognitive decline (69.5%), respectively.

The characteristics between BD patients with (*N* = 21) and without (*N* = 89) longitudinal cognitive decline are presented in Table 1. Only two participants had psychiatry comorbidity with cluster B personality disorders. None of the participants was comorbid with schizophreniform disorder, delusional disorder, anxiety disorders, alcohol or illegal substance use disorders, organic mental disorders, obsessive-compulsive disorder, attention deficit/hyperactivity disorder or autistic spectrum disorders. The mean follow-up period from index IQ to current BAC-A assessment (T2 to T3) was 6.5 years, which showed no difference between the two groups. Compared with patients without cognitive decline, those with cognitive decline were younger, experienced more manic episodes, especially episodes with psychotic features, more number of admissions, and higher hospitalization duration. In addition, the cognitive decline group revealed using higher dose of mood stabilizer and less dose of antidepressant at time point T3 than those without cognitive decline. No significant differences were observed in variables such as physical comorbidities, duration of illness, age of onset, or index IQ measurement between the two groups.

**Table 1:** Sociodemographic and clinical characteristics of BD patients with and without cognitive decline.

	**Without cognitive decline (*N =* 89)**	**With cognitive decline (*N =* 21)**	***P*-value**
Gender Male, n (%)	34 (38.20)	10 (47.62)	0.428
Marriage: married or lived together, n (%)	34 (38.20)	4 (19.05)	0.097
Job: regular job, n (%)	23 (25.84)	4 (19.05)	0.515
With family psychiatry history, n (%)	48 (53.93)	13 (61.90)	0.509
Smoking, n (%)	15 (17.24)	3 (14.29)	0.999
Alcohol use habit, n (%)	1 (1.14)	1 (4.76)	0.443
Current physical comorbidity, n (%)	18 (20.69)	4 (19.05)	0.999
Seasonality, n (%)	30 (33.71)	6 (28.57)	0.652
Age (yr)^a^	50.35 ± 13.49	43.81 ± 10.49	0.040^*^
Onset of age (yr)^a^	27.86 ± 12.05	22.81 ± 8.48	0.072
Education years^a^	12.99 ± 3.16	12.95 ± 2.73	0.962
Index full IQ	93.39 ± 16.08	88.75 ± 19.87	0.460
Performance IQ	90.00 ± 18.06	81.83 ± 20.63	0.235
Verbal IQ	96.91 ± 15.45	97.67 ± 19.9	0.902
Follow up duration (month)^a^	57.39 ± 49.34	110.00 ± 88.45	0.076
Duration of illness (year)	22.44 ± 11.08	21.71 ± 10.03	0.784
Total episode density^a^	0.40 ± 0.27	0.51 ± 0.24	0.070
Manic episode density^a^	0.26 ± 0.21	0.40 ± 0.24	0.013^*^
Major depressive episode density^a^	0.13 ± 0.15	0.10 ± 0.13	0.413
Episode with psychotic features density^a^	0.15 ± 0.16	0.29 ± 0.25	0.023^*^
Maximum of free interval (month)^a^	91.38 ± 70.27	67.24 ± 49.04	0.140
Number of suicide attempt^a^	0.77 ± 1.60	0.71 ± 0.85	0.817
Days of hospitalization^a^	191.48 ± 195.97	354.38 ± 201.68	0.001^*^
Number of admission^a^	5.13 ± 5.31	8.00 ± 5.29	0.028^*^
Index body mass index, BMI^a^	22.53 ± 5.29	24.01 ± 5.21	0.258
**Current psychoactive agents (DDD)**			
First-generation antipsychotics^a^	0.06 ± 0.17	0.16 ± 0.48	0.357
Second-generation antipsychotics^a^	3.29 ± 4.48	6.52 ± 7.52	0.071
Mood stabilizers^a^	0.51 ± 0.35	0.73 ± 0.46	0.018^*^
Antidepressants^a^	0.07 ± 0.28	0.00 ± 0.00	0.016^*^
Benzodiazepines^a^	0.50 ± 0.75	0.47 ± 0.62	0.829

* *p < 0.05;*

a *Assessed using Wilcoxon rank sum test*.

*IQ, intelligence quotient; DDD, defined daily dose*.

### Functional Outcomes and BAC-A in BD Patients With Cognitive Declines

No significant differences were observed in current functional outcomes assessed using WHODAS 2.0 between BD patients with or without cognitive decline. Table 2 shows the IRT scoring of the groups; a higher score indicated more severity of subjective impairment, that is, greater disability. We noted that common functional impairments in the chronic phase of BD were observed in both groups, regardless of presence of cognitive decline. Even for the most preserved functional domain (self-care with IRT score 14.0), patients showed impairment that was the last 30% in the population percentile when compared with norm data (39). In addition, there were no difference from HDS, YMRS and childhood trauma experience between two groups. For cognitive profiles, each patient's performance in individual tests was compared with an age- and gender-matched Taiwan norm to calculate the z-score for six traditional neurocognitive domains (36). As observed in BAC-A assessment, the cognitive decline group showed significant worsening of all six neurocognitive domains, not limited to specific domains. Moreover, APT indexes indicated the different emotional inhibitions between the groups (Table 2).

**Table 2:** Mood status, functional assessment and cognitive profiles at follow-up in BD patients with and without cognitive decline.

	**Without** ** cognitive decline (*N =* 89)**	**With** ** cognitive decline (*N =* 21)**	***P*-value**
WHODAS2.0 Total score	31.15 ± 18.57	23.90 ± 11.41	0.258
D1 cognition	26.54 ± 18.43	26.00 ± 13.7	0.934
D2 walk^a^	19.44 ± 27.87	17.60 ± 6.65	0.284
D3 self-care^a^	13.11 ± 17.54	14.00 ± 6.99	0.730
D4 along with others	35.19 ± 22.17	28.50 ± 15.81	0.390
D5-1 housekeeping^a^	35.26 ± 32.76	27.00 ± 20.58	0.110
D5-2 job & learn^a^	23.00 ± 29.56	25.25 ± 10.50	0.285
D6 social^a^	32.93 ± 23.41	25.60 ± 14.61	0.364
HDS score^a^	2.40 ± 1.95	2.81 ± 1.94	0.393
YMRS score^a^	2.72 ± 2.27	2.86 ± 2.33	0.803
CTQ total^a^	61.47 ± 7.80	64.5 ± 19.39	0.675
BAC-A composite score^a^	−0.83 ± 1.33	−4.51 ± 1.63	<0.0001^*^
Verbal memory^a^	−0.18 ± 1.37	−2.69 ± 2.52	0.0002^*^
Motor speed^a^	−1.32 ± 1.19	−3.34 ± 1.56	<0.0001^*^
Working memory^a^	−0.37 ± 0.98	−1.99 ± 1.51	<0.0001^*^
Verbal fluency	−0.20 ± 1.10	−1.44 ± 0.71	<0.0001^*^
Attention and processing speed	−0.92 ± 1.31	−2.62 ± 0.92	<0.0001^*^
Executive function^a^	−0.08 ± 1.15	−2.39 ± 2.11	<0.0001^*^
**Affective processing tests**			
AIT: total non-affective words^a^	13.36 ± 4.45	10.95 ± 4.18	0.026^*^
AIT: total affective words	10.7 ± 4.47	8.48 ± 4.49	0.043^*^
AIT: cued non-affective words^a^	3.69 ± 2.01	3.10 ± 2.19	0.238
AIT: cued affective words^a^	5.38 ± 2.00	4.19 ± 1.47	0.012^*^
DR: correct non-affective words^a^	17.85 ± 2.08	16.29 ± 2.90	0.028^*^
DR: correct affective words^a^	16.94 ± 2.39	15.95 ± 3.41	0.219
DR: non-affective false alarms^a^	2.15 ± 2.08	3.71 ± 2.90	0.028^*^
DR: affective false alarms^a^	3.06 ± 2.39	4.05 ± 3.41	0.219
EII (emotion inhibition index)	−79.38 ± 23.28	−66.62 ± 23.51	0.026^*^

* *p < 0.05;*

a *Assessed using Wilcoxon rank sum test*.

*HDS, Hamilton depression scale; YMRS, Young mania rating scale; CTQ, Childhood Trauma Questionnaire; AIT, affective interference test; DR, delayed recognition; EIT, emotion inhibition test*.

### Risk Factors for Cognitive Decline and Decline Curve in Patients With BD

We used univariate and multivariable logistic regression to analyze the independent risk factors for cognitive decline in BD, and results are displayed in Table 3. Because the extreme low dose of antidepressants used among BD patients (mean DDD was 0.00 in cognitive decline group and 0.07 in without cognitive decline group), we were not able to assess the effects of antidepressants DDD on cognitive decline in regression models. In multivariable logistic regression models controlling for age and gender, we found that density of episodes with psychotic features during the disease course (odds ratio [OR] 25.21, 95% CI 2.15–259.61, *p* = 0.010) and DDD of mood stabilizers (OR 3.86, 95% CI 1.03–14.89, *p* = 0.049) were independent risk factors for cognitive decline.

**Table 3:** Risk factors for cognitive decline in BD using univariate and multiple logistic regression analyses.

	**Univariate**				**Multivariable**		
	**OR**	**(95% CI)**	***p*-value**		**OR**	**(95% CI)**	***p*-value**
Age						
≧40	1.00				1.00		
>40	0.55	(0.20–1.46)	0.231		0.68	(0.23–1.98)	0.457
Gender						
Female	1.00				1.00		
Male	1.47	(0.57–3.83)	0.430		0.99	(0.34–2.97)	0.996
Manic episode density	10.02	(1.35–74.57)	0.024^*^				
Episode with psychotic features density	26.90	(2.44–297.13)	0.007^*^		25.21	(2.15–259.61)	0.010^*^
DDD of Mood stabilizers	4.24	(1.23–14.67)	0.022^*^		3.864	(1.03–14.89)	0.049^*^

* *p < 0.05*.

*Multivariable regression using stepwise selection for age, gender, manic episode density, psychotic episode density and DDD (defined daily dose) of mood stabilizers. By the selection, final model including age, gender, episode with psychotic features density and DDD of mood stabilizers*.

**Figure 1:**
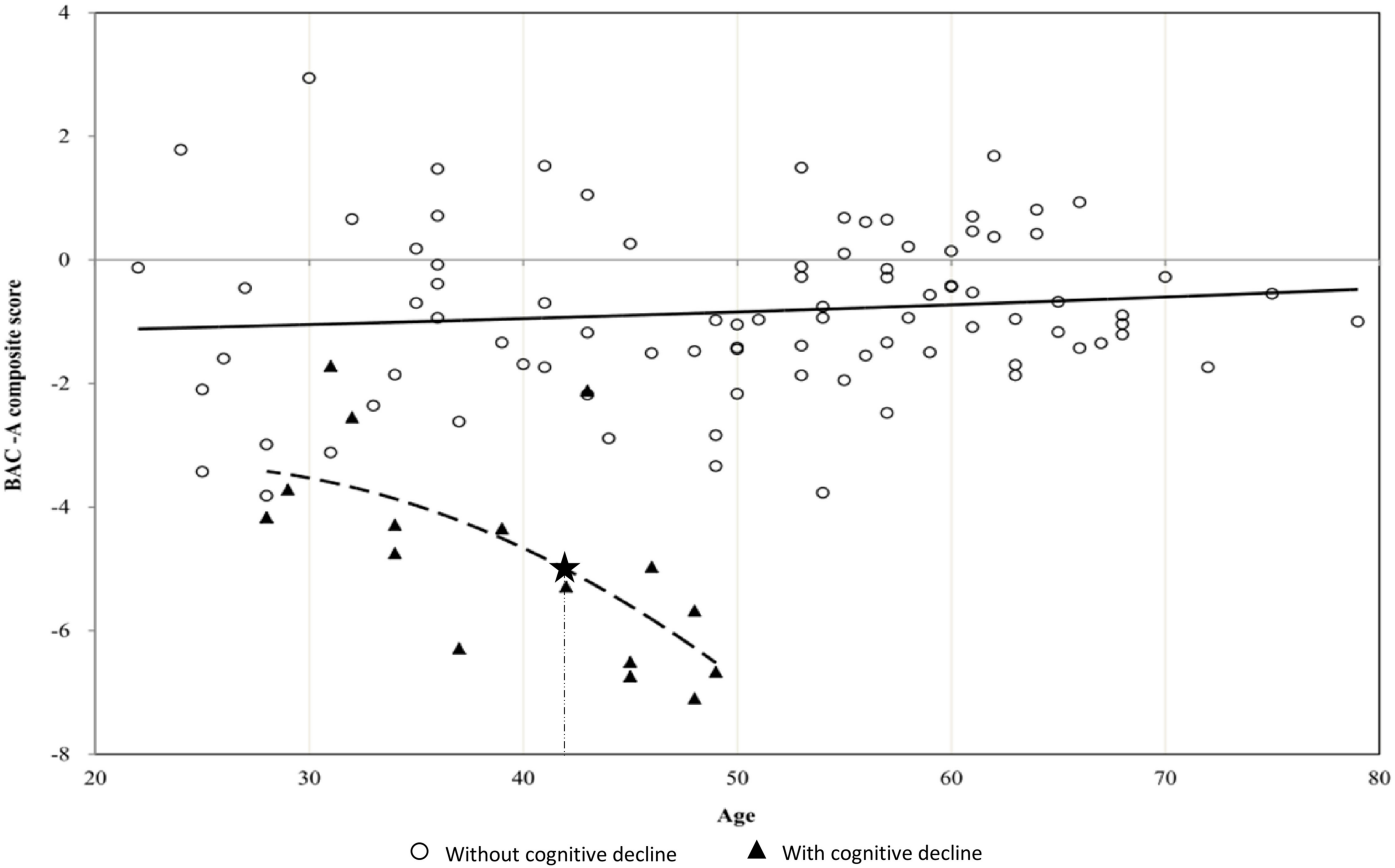
Scatterplot between age and BAC-A composite score in BD patients with or without cognitive decline. The figure depicts steeper decrease in BAC-A composite score with hinge points (asterisk mark) at the age of 42 years in BD patients with cognitive decline. The slope of dotted curve showed −0.276 SD per year after age 42.

Figure 1 depicts a steeper decline in BAC-A composite score in patients with cognitive decline compared with those without cognitive decline. In addition, MARS yielded a hinge point at the age of 42 years for the cognitive decline group, and their BAC-A composite score steeply worsened by 0.276 SD per year after this age.

### Correlation Between Cognitive and Functional Domains

**Figure 2:**
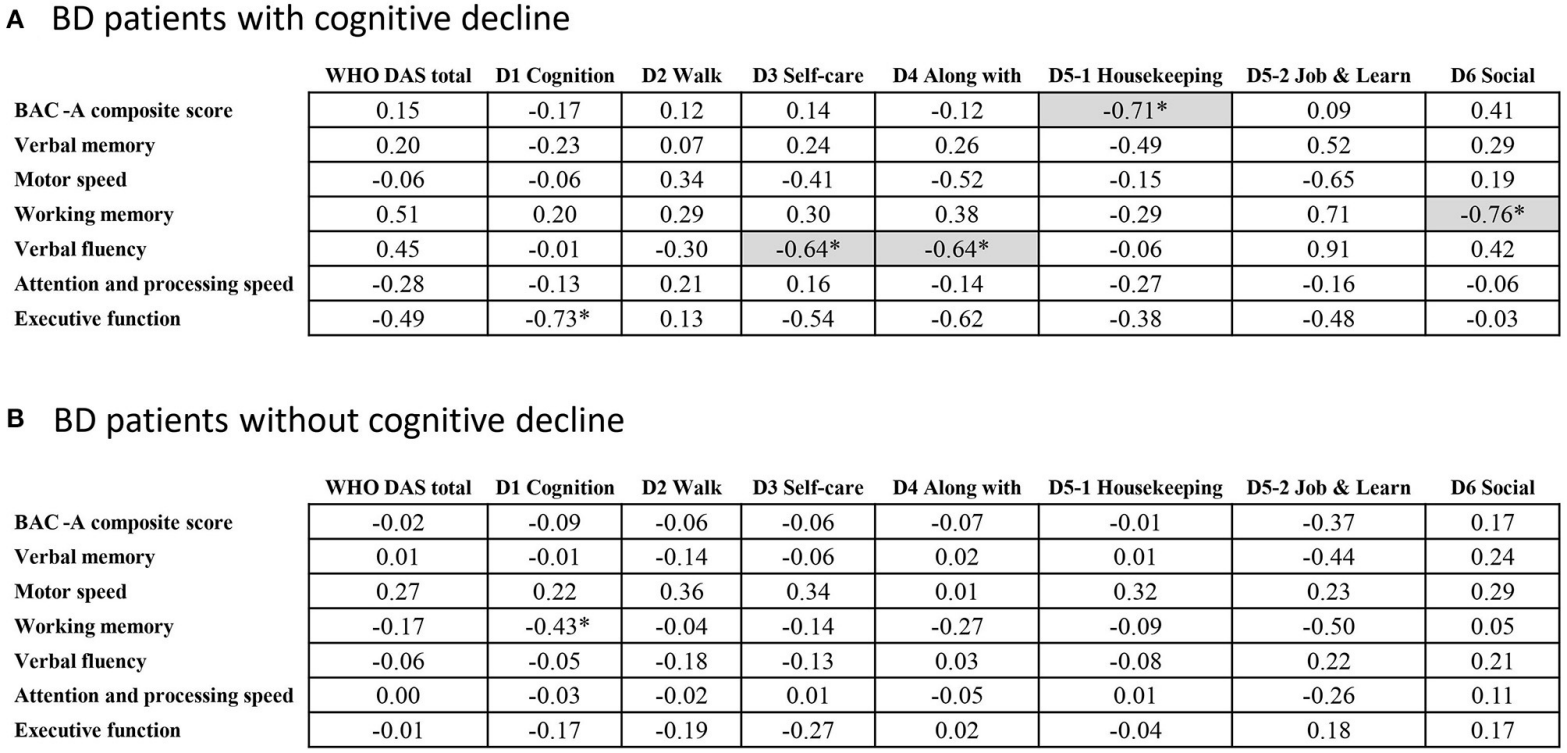
Pearson correlations between cognitive and functional domains in BD. *p < 0.05; Gray base indicating the significance after adjusting the effect of age.

Figure 2 demonstrated correlations between cognitive and subjective functional domains in patients with (Figure 2A) and without cognitive decline (Figure 2B). We evaluated whether there was the same trend of correlations for both groups of BD, where better cognitive scores were correlated to lower subjective dysfunction. We found that in the group without cognitive decline, no significant correlation was found between functional and cognitive domains after adjusted for age. In contrast, the cognitive decline group showed strong links between various cognitive domains and daily functions (*r* ranging from 0.64 to 0.76). For example, the range of impaired functions, such as self-care and getting along with other people were negatively correlated with verbal fluency. Working memory and composite score of cognition also have negative correlation with subjective social and housekeeping function, respectively. We tested differences for the aforementioned four pairs of correlation coefficients between the two groups using Z test, and the correlation differences reached significance (*P* < 0.05). In this part, the different correlation patterns of the two groups suggested the heterogeneous effects on daily function by their longitudinal cognitive profiles.

## Discussion

To the best of our knowledge, this retrospective cohort study is the first to provide a comprehensive picture of BD patients with cognitive decline through long-term follow-up, and the results suggested the existence of subgroups with different cognitive trajectories. We classified our patients into two groups according to presence or absence of longitudinal cognitive decline and correlated cognitive profiles to their daily dysfunctions. We revealed that more number of episodes with psychotic features and current high DDD of mood stabilizers were risk factors to be associated with cognitive decline. Furthermore, we demonstrated a hinge point at the age of 42 years with steeper decline of cognition in the cognitive decline group.

In our study, the picture of BD patients with longitudinal cognitive decline shows wide-range of domains of impairment, rather than on only some specific cognitive domains, which is consistent with most previous cross-sectional studies (44). Moreover, the magnitude and proportion of cognitive dysfunction reported in previous cross-sectional studies, which used BAC-A to assess cognitive function in BD, is similar to that obtained by combining the three subgroups in the present study (35, 45). Therefore, our findings, showing longitudinal changes of BD are robust as suggested by those of previous cross-sectional literature. More specifically, our findings of longitudinal cognitive heterogeneity in a small subset of patients with BD are important and can partially explain the diverse and inconsistent findings for clinical characteristics and risk factor identification in the literature. In our study, the longitudinal cognitive decline group comprised around 16.4% of our sample, and this group may be established as a specific subtype of BD. Such longitudinal cognitive profiles can potentially facilitate further genetic and biological studies and help clinicians develop more effective and personalized intervention strategies (46, 47). As we had suggested, the heterogeneity of the cognitive trajectory conformed to the progress of illness in clinical manifestation staging models including cognitive deterioration (48). Our findings revealed that cognitive decline varies from one patient to another, with decline in certain patients while others remain stable. Conversely, we may say that some patients maintained relatively stable cognitive function might be cognitive reserve, which reflects the partial capacity of the brain to endure neuropathology and minimize clinical cognitive deficits (49). Our findings also suggested the paradigms of cognitive impairment in BD as persistently stable or progressive are not exclusive or this may partially overlap (50). The verification of heterogeneity of longitudinal cognitive change in small part of BD samples further comprehend the different faces of cognition in this disorder.

The risk factors identified for cognitive decline in BD suggest the converging evidence that patients with BD show cognitive impairment related to the clinical course. However, most previous studies have not considered potential longitudinal heterogeneity and have thus compared all BD patients together, leading to masking risk factors findings in subgroups of cognitive change over time in BD (51–54). Recently, a cohort study that assessed longitudinal cognitive changes in BD showed that a higher number of manic episodes is associated with a decrease in global cognition as well as working memory and visual memory (51). Our findings were consistent with this study and further implied the association of cognitive impairment with episodes with psychotic features (55, 56). Another risk factor noted in this study is the dose of mood stabilizer used at the BAC-A assessment. It is clear that higher dose of lithium or anticonvulsants has varying effects on cognitive function (57, 58); however, we cannot conclude the causal effect of dose of mood stabilizer to the risk of cognitive decline from this findings, as the dose recorded at T3 of the study and there may exist some confounding by indications with the patients presence of more manic episodes and cognitive deficits at the same time.

Although our findings implied the associations between episodes with psychotic features and cognitive decline in patients with BD, very little is known about the underlying mechanism why some patients with BD develop significant cognitive decline while others remain cognitively intact. There may exist unmeasurable confounding factors in this cohort. The association of psychotic features with cognitive decline in BD was corroborated by studies from psychosis populations who have been found to exhibit smaller total brain volume (59) and reduced functional connectivity in frontoparietal control network (60). In addition, meta-analyses pooling inconsistent results from each individual study revealed findings of reduced white matter integrity and volume in BD (61, 62). We need large sample size in future studies to tackle the heterogeneity issue for cognitive impairment. In addition, we recommend that further genetic risk or biomarker studies should be conducted in this subgroup of patients. Therefore, the precise intervention can apply earlier in this subgroup, such as active relapse prevention or cognitive remediation. Moreover, the subgroup of BD patients with cognitive decline showed steeper changes after the age of 42 years compared with those without cognitive decline. The hinge point of age 42 in cognitive decline group is on average 20 years after the disease onset, implying that long-term follow-up is needed for detecting substantial cognitive change. On the other hand, there is time to design and implement intervention strategies to alleviate potential cognitive decline. Our results are consistent with the findings that there was accelerated aging in executive functions in BD (63), therefore, we suggested that clinicians should pay attention to patients' cognitive function in their early middle age.

Psychosocial function is a person's ability to perform activities of daily living and to be involved in meaningful interpersonal relationships. As per our results, patients with BD have difficulties in several areas of function during their remitted status. These results are consistent with those of other studies demonstrating that most patients with BD have functional difficulties assessed by Functioning Assessment Short Test (FAST) (64–66) and WHODAS 2.0 (67). However, our findings suggested no difference of functional outcomes in patients with and without cognitive decline assessed by WHODAS 2.0. There may be possible floor effect of this subjective assessment for this population, as both groups exhibit poor functional scores. Thus, we are not able to detect meaningful differences between the two subgroups with relatively small samples. We used the WHODAS 2.0 for evaluating our patients, rather than disease-specific questionnaires, such as FAST, because the former disability assessment is more informative by comparing with general norm or other diseases. Further results from the partial correlation analysis between cognitive and functional domains, controlling for age, indicated that the cognitive profile was still correlated with some functional domains mainly in the group of BD patients with cognitive decline. These results are in line with those of cross-sectional studies showing that the cognition-function relationship may be weaker among patients without cognitive deficits than among those with cognitive impairment (68). The distinct correlation results among patients with BD also responded to those of a previous study conducted by Sole et al. They suggested the more robust correlation between the poor function group of BD to their cognitive function and smaller correlations in less functional impaired BD (69).

Several limitations should be considered of this study. First, our study sample was recruited from a tertiary psychiatry hospital where patients have more severe degree of illnesses. This potentially biased sample may limit the generalizability of our findings to the whole BD population, especially the proportion with cognitive decline. Moreover, we cannot exclude the possibilities of recall bias in reporting clinical related information, such as lifetime psychiatric comorbidities. Nevertheless, we had checked medical charts to verify records in comorbidities and medication to minimize such bias. Second, without a healthy control group, cognitive decline in patients should be viewed as evidence of relative cognitive decline. Third, as mention before, we used different measurements for three time-points of cognitive assessment. Despite of this limitation, all of the raw scores have been transformed into standardized Z scores according to Taiwan's norm data. Forth, despite the correlation patterns between functional and cognitive domains showed significant differences between the two subgroups (Figure 2), such differences required further validation, as well as to minimize potential confounding effects from other variables. Furthermore, the restricted sample size limited the power to examine interaction effects among potential variables, such as interaction of disease course and medications. Fifth, WHODAS 2.0 is an interviewer-administered tool assessing subjective disability; a combination of WHODAS 2.0 administered from caregivers or other objective measures may provide a more comprehensive and accurate outcome picture for BD patients with and without cognitive decline. Sixth, we have no data about inter-rater reliability in relation for measuring IQ and function. Finally, the dichotomous categorized definition about cognitive decline (difference more than 2SD) in our study is arbitrary. However, the potential of misclassification is non-differential and bias the odds ratio toward the null, which suggested the robustness of our findings. In the current study, we adjusted the medication effect only at time point of T3, the full picture of medication usage during the disease course cannot be easily captured. In addition, due to the extreme low dose of antidepressants used in our samples, we're not able to evaluate the effect of antidepressants use on cognition decline. Further detailed data collection in larger samples and advanced methodology might be helpful to overcome these issues. On the other hand, with a retrospective cohort design, we have the strength to confirm the diagnosis as bipolar disorder through the disease course. Future prospective studies should be cautious about including patients that may change diagnosis from BD to schizoaffective disorder or schizophrenia during follow-up period. Further investigations in the field are necessary for uncovering the underlying mechanisms linking risk factors, cognitive decline, and functional outcomes.

In conclusion, our results specify and characterize cognitive heterogeneity in BD longitudinally, which may facilitate further genetic and biological studies to define more valid BD subtypes to reveal the underlying mechanisms. We identified risk factors for cognitive decline and therefore suggested aggressive relapse prevention. The heterogeneity of cognitive decline in BD should be considered, thus individualized intervention for patients with BD could be applied in the future.

## Data Availability Statement

The original contributions presented in the study are included in the article/Supplementary Material, further inquiries can be directed to the corresponding author/s.

## Ethics Statement

The Research Ethics Committee of Taipei City Hospital approved our study (TCHIRB-1-703103). The patients/participants provided their written informed consent to participate in this study.

## Author Contributions

W-YC performed conceptualization, funding acquisition, project administration, and writing the original draft. M-CH, S-KL, H-CL, P-YC, and CC performed investigation and resources. Y-CL, S-HW, and C-EC done software analysis and data curation. C-JK performed the suggestion for investigation, resources, and data curation. P-HK was major for supervision, methodology, and reviewing the manuscript. All authors read and approved the final manuscript.

## Conflict of Interest

The authors declare that the research was conducted in the absence of any commercial or financial relationships that could be construed as a potential conflict of interest.
