# Supplementary material for: The Heterogeneity of Longitudinal Cognitive Decline in Euthymic Bipolar I Disorder With Clinical Characteristics and Functional Outcomes
Source: Front Psychiatry. 2021 Jul 21;12:684813. doi: 10.3389/fpsyt.2021.684813 (PMC8335543; doi:10.3389/fpsyt.2021.684813)
Supplement: Supplementary file 1 [file Data_Sheet_1.PDF]

Supplementary Figure 1. Time points assessing cognitive function in current study

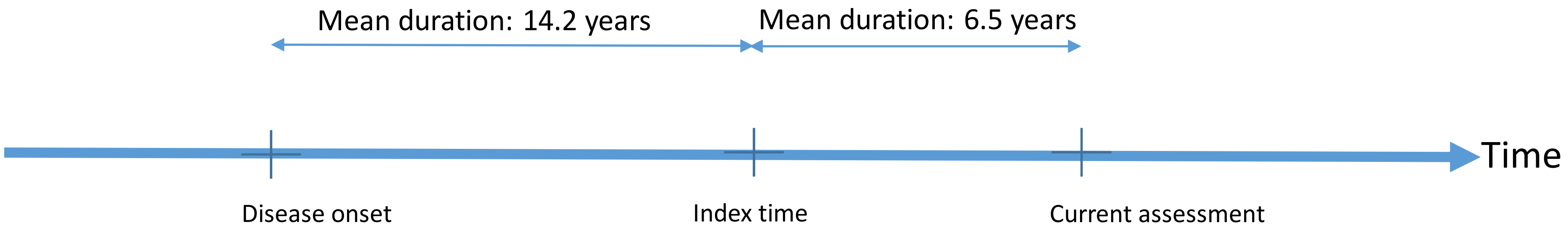

Time point	T1	T2	T3
Measurement	Estimated premorbid IQ *	WASI IQ	BAC-A

*Estimated premorbid IQ based on age, gender, occupational status and years of education from Taiwan norm data
Abbreviations: WASI, Wechsler Adult Intelligence Scale; IQ, Intelligence Quotient ; BAC-A, Brief Assessment of Cognition in Affective Disorders
